# Control and evaluation of a humanoid robot with rolling contact joints on its lower body

**DOI:** 10.3389/frobt.2023.1164660

**Published:** 2023-10-16

**Authors:** Seung Hyeon Bang, Carlos Gonzalez, Junhyeok Ahn, Nicholas Paine, Luis Sentis

**Affiliations:** ^1^ Department of Aerospace Engineering, University of Texas at Austin, Austin, TX, United States; ^2^ Department of Mechanical Engineering, University of Texas at Austin, Austin, TX, United States; ^3^ Apptronik, Inc., Austin, TX, United States

**Keywords:** rolling contact joints, whole-body control, humanoid robots, legged robots, humanoid system integration

## Abstract

In this paper, we introduce a new teen-sized humanoid platform dubbed DRACO 3, custom-built by Apptronik and altered for practical use by the Human Centered Robotics Laboratory at The University of Texas at Austin. The form factor of DRACO 3 is such that it can operate safely in human environments while reaching objects at human heights. To approximate the range of motion of humans, this robot features proximal actuation and mechanical artifacts to provide a high range of hip, knee, and ankle motions. In particular, rolling contact mechanisms on the lower body are incorporated using a proximal actuation principle to provide an extensive vertical pose workspace. To enable DRACO 3 to perform dexterous tasks while dealing with these complex transmissions, we introduce a novel whole-body controller (WBC) incorporating internal constraints to model the rolling motion behavior. In addition, details of our WBC for DRACO 3 are presented with an emphasis on practical points for hardware implementation. We perform a design analysis of DRACO 3, as well as empirical evaluations under the lens of the Centroidal Inertia Isotropy (CII) design metric. Lastly, we experimentally validate our design and controller by testing center of mass (CoM) balancing, one-leg balancing, and stepping-in-place behaviors.

## 1 Introduction

Dynamic behaviors for legged robots require extensive sensing and actuation, as well as high-performance control in terms of efficiency, range of motion, speed, and accuracy, among other factors. At the mechanical and real-time control levels, several important matters need to be considered. On the one hand, efficient transmissions with low friction/stiction and low backlash are desired. In addition, mechanical designs, capable of achieving a wide range of motion (RoM), are important and non-trivial to achieve. On the other hand, these types of mechanisms come at the expense of an increase in complexity of the controllers needed to exploit the potential of the high-dimensional humanoid system.

Humanoids have often employed collocated actuators (motors directly located at each joint) because of their simplicity in design (Park et al., 2007; Radford et al., 2015). However, the performance of these robots degrades when using simplified models for planning because of model discrepancy caused by the heavy distal mass on their legs (Sim and Ramos, 2022). Due to this problem, their design has shifted in favor of proximal actuation (placing heavy motors near the torso) to reduce the limbs’ distal mass for dynamic maneuvers. A frequently explored technique to achieve proximal actuation in legged robots has been through the use of cable-driven transmission systems, mainly due to the high torque/power density arising from their lightweight and effective power transmission capabilities (Mazumdar et al., 2017; Hwangbo et al., 2018; Liu et al., 2022). Additionally, this kind of transmission is more efficient because the mechanical losses due to friction are small and, thus, provide higher torque transparency.

**FIGURE 1:**
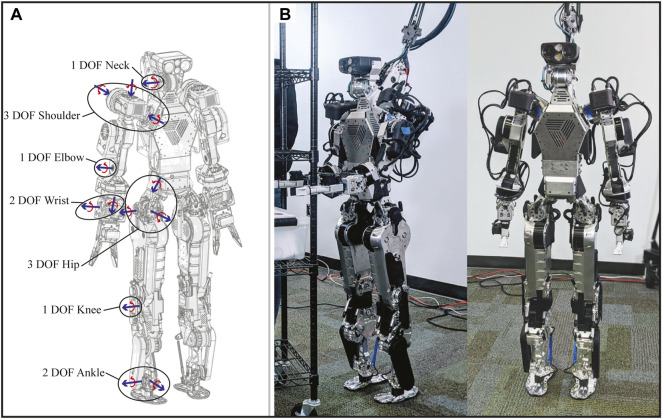
DRACO 3 humanoid. **(A)** Wireframe of DRACO 3 showing the degrees of freedom on the right side of the robot and their respective axis of rotation. **(B)** DRACO 3 standing at full height.

In this vein, DRACO 3 (Figure 1) was designed bearing a cable-based drive system in mind for proximal actuation. In addition, rolling contact joints (RCJs) are employed to enhance their RoM. Although RCJs have been widely used in other fields, such as in lower extremity exoskeletons, to reduce misalignment between the human’s and exoskeleton’s joints (Beil and Asfour, 2019; Wang et al., 2018) and in robotic fingers to achieve large RoM as well as to decrease internal friction (Boisclair et al., 2021), they have not been widely adopted in humanoids due to their already high mechanical complexity and intricate control design. In our case, the hip flexion/extension and knees of DRACO 3 are designed as RCJs, enabling proximal actuation and an increased RoM at the expense of mechanical and control complexity.

Current methods to control complex robots such as humanoids often use whole-body controllers (WBCs) (Sentis et al., 2010; Kim et al., 2020; Lee et al., 2022), which compute joint-level commands to achieve desired operational space tasks while in contact. These types of controllers are often designed by considering a robot model along with multiple physical and environmental constraints (e.g., surface friction). In addition, any complexities in the joint mechanisms must be taken into account either within or outside the WBC module. Ahn et al. (2021) presented a rich literature survey on different types of WBCs. In this work, we extend the analyses of Ahn et al. (2021) and IHMC Robotics (2018) by incorporating new types of mechanical constraints and exploit our frameworks’ high flexibility to manage tasks and contacts and to ease robot controller tuning.

Previous works such as Collins (2003), Wang et al. (2018), Beil and Asfour (2019), and Boisclair et al. (2021) have studied the control of complex joint mechanisms (e.g., RCJs) in an isolated manner by developing a high-fidelity characterization of the transmission. The presence of such joint mechanisms in high-dimensional robotic systems is emerging, and so are the control architectures and WBCs that perform effectively using these types of mechanisms. For instance, series and parallel linear actuators have been incorporated into WBCs either through transmission mappings (Radford et al., 2015; Englsberger et al., 2018) or by defining the corresponding optimization problem in the actuation space (Mronga et al., 2022). Another popular mechanism used in legged robots is the four-bar linkage, e.g., MiniHyQ (Khan et al., 2015) and IHMC’s Nadia humanoid (IHMC Robotics, 2018). Although the latter also makes use of a WBC taking into account a four-bar linkage mechanism, here, we show detailed derivations to integrate RCJs into a WBC. To the best of our knowledge, our work represents the highest degree of self-containment among the literature (e.g., integration of the RCJ into a WBC framework for humanoid robots). Furthermore, we argue this work represents a more detailed mathematical representation and integration than the existing work.

Although there exist many works addressing weighted-task-prioritized WBC for legged robots (Koolen et al., 2016; Caron et al., 2019; Ramuzat et al., 2021), they rarely discuss their handling of the intricate mechanical transmissions that the robots contain. In particular, it is non-trivial to model task kinematics for complex mechanical structures like DRACO 3. In contrast, we provide more details and tools to incorporate complex transmissions into the kinematic models, such as RCJ transmissions. In addition, we provide valuable details about the implementation of WBCs, which are sometimes overlooked in other works (Koolen et al., 2016; Caron et al., 2019; Ramuzat et al., 2021). These implementation details have proven effective on hardware, including but not limited to careful selection of tasks, their corresponding Jacobians, and optimization parameters. Shen et al. (2022) discussed practical guidance for WBC implementation, such as controlling the ankle position instead of some point on the bottom foot (task selection). However, their strategy is limited to line-feet miniature bipedal robots. Similarly, Kim et al. (2020) and Jorgensen (2020) chose to control the base height instead of the center of mass (CoM) height (task selection) and used a modified swing foot Jacobian (Jacobian selection) with zeros on the floating base degrees of freedom (DOFs). However, their applicability was only tested on a line-feet robot with light legs. Here, we experimentally validate that controlling the base height and using the modified swing foot Jacobian remain effective for a teen-sized humanoid robot with relatively large leg mass. Additionally, we provide guidance on parameter selection for the reaction wrench regularization of planar contact, which has not been found in the previous literature.

In summary, the main contributions of this paper are as follows: 1) an introduction to the new humanoid robot DRACO 3, custom-built by Apptronik, where we highlight design modifications that we have performed at the Human Centered Robotics Laboratory to increase the durability, performance, and practical use of the robot; 2) an extension to our previous WBC framework to take into account RCJs by means of internal constraint inclusions; 3) a detailed implementation of the proposed WBC for practical guidance on hardware implementation for humanoids with a relatively large leg mass; 4) an analysis on the centroidal properties of the DRACO 3 robot showing its comparison with other known and relatively large humanoids; and 5) a set of initial proof-of-concept tests showing DRACO 3’s performing force disturbance rejection, lateral swaying, balancing while squatting, one-leg balancing, and stepping-in-place behaviors to validate our design models as well as the integration of our extended WBC into DRACO 3.

## 2 System overview

In this section, we introduce the overall actuation mechanisms of DRACO 3, placing greater emphasis on the hip and knee mechanisms, which make up the most complex parts of the robot. We further explain how DRACO 3 achieves a large range of motion with minimal backlash throughout its movements while also favoring proximal actuation.

### 2.1 Robot design

**FIGURE 2:**
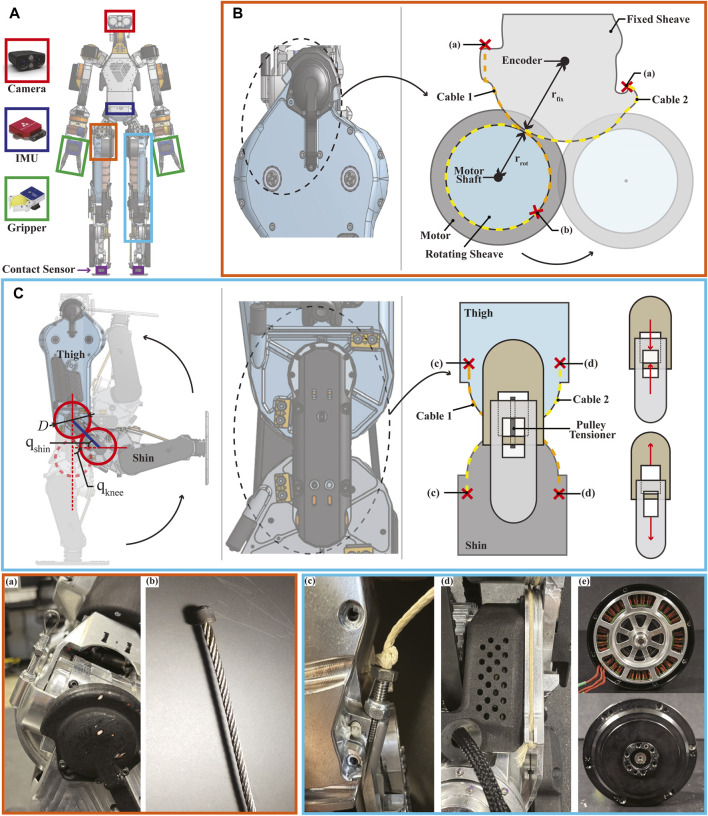
Cable-driven actuation on lower limbs. **(A)** Mechatronic components in DRACO 3: the camera, the IMU, the grippers, and the contact sensors are the Carnegie Robotics MultiSense S7, VectorNav VN-100, SAKE Robotics EZGripper, and Apptronik’s strain gauge, respectively. **(B)** Hip pitch actuation: lateral view of the hip pitch joint showing cable routing (---) and the location of the terminations (×) of the stainless steel cables. Crimped termination and soldered termination are shown in figures (a) and (b), respectively. **(C)** Knee actuation: snapshots of leg configurations in sequence when q_knee_ is 0°, 90°, and 180° (left). Note that because of the large knee ROM, the lower leg folds flat against the thigh. Lateral view of the knee showing cable routing (---), the location of its cable terminations (×), and an adjustable tensioning mechanism (right). Estar stopper knot termination is adopted as shown in figures (c) and (d). The gear ratio of the knee motor was up-sized to be 16:1 instead of the original 9:1, without altering the original motor outer dimensions, as shown in figure (e).

DRACO 3 stands 1.35 m tall, weighs 39 kg, and has a RoM similar to that of a human (Roaas and Andersson, 1982; Doriot and Wang, 2006) in its actuated DoF: neck pitch, 6-DoF arms, and 6-DoF legs. The joint configuration shown in Figure 1A is used by the low-level controller, and its RoM, speed, and torque limits (defined in these coordinates) are shown in Table 1. Table 2 compares the lower limbs’ design characteristics with those of adult-sized humanoid robots. In addition, DRACO’s proprioceptive and exteroceptive sensors are illustrated in Figure 2A.

**TABLE 1:** Joint, velocity, and torque limits of DRACO humanoid in the generalized coordinates.

Joint name	RoM (deg); [min, max ]	Max; Torque (nm)	Max; Velocity (rad/s)
Neck pitch	[−30, 75]	8	76
Shoulder roll	[0, 120]	18	47
Shoulder pitch	[−89, 89]	18	47
Shoulder yaw	[−130, 40]	18	47
Elbow pitch	[−120, 5]	10	59
Wrist roll	[−90, 90]	12	71
Wrist pitch	[−90, 90]	8	76
Hip roll	[−15, 45]	56	5
Hip pitch	[−87, 30]	59	36
Hip yaw	[−50, 50]	44	8
Knee pitch	[−10, 175]	36	15
Ankle roll	[−45, 15]	30	11
Ankle pitch	[−90, 60]	44	8

**TABLE 2:** Comparison of lower limbs’ design characteristics of various humanoid robots.

Robot	Height (m)	Total mass (kg)	Leg mass (kg)	Leg DOF	RoM (deg); [min, max ]	Max torque (nm)	Max velocity (rad/s)
Atlas	1.88	150	18.11	Hip roll	[−30, 30]	530	12
				Hip pitch	[−92, 38]	840	12
				Hip yaw	[−45, 10]	275	12
				Knee pitch	[0, 135]	890	12
				Ankle roll	[−46, 46]	45	12
				Ankle pitch	[−57, 40]	92	12
Valkyrie	1.87	129	24.76	Hip roll	[−32, 27]	350	7
				Hip pitch	[−139, 93]	350	6
				Hip yaw	[−63, 24]	190	6
				Knee pitch	[−5, 118]	350	11
				Ankle roll	[−23, 23]	205	11
				Ankle pitch	[−53, 37]	205	11
Hubo2 Plus	1.3	42	10.23	Hip roll	[−28, 28]	10	1
				Hip pitch	[−85, 92]	10	1
				Hip yaw	[−90, 90]	10	1
				Knee pitch	[−4, 149]	10	1
				Ankle roll	[−11, 11]	10	1
				Ankle pitch	[−74, 97]	10	1
DRACO 3	1.35	39	9.59	Hip roll	[−15, 45]	56	5
				Hip pitch	[−87, 30]	59	36
				Hip yaw	[−50, 50]	44	8
				Knee pitch	[−10, 175]	36	15
				Ankle roll	[−15, 45]	30	11
				Ankle pitch	[−90, 60]	44	8

The upper body is composed of direct and quasi-direct drives for mechanical simplicity and robustness using mostly the off-the-shelf actuators. Each arm is composed of five off-the-shelf motors, an Apptronik’s motor on the wrist pitch, and a gripper. On the other hand, the design of the lower body is more elaborate. The hip pitch and knee joints are cable-driven joints. Despite both of them being cable-driven, the hip is effectively a revolute joint, while the knee is a rolling contact joint. The rolling joint combined with the cable-driven mechanism leads to a reduced total and distal leg mass, which helps regulate the impact force more easily by reducing leg dynamics that complicate the swing-leg control (Wensing et al., 2017) and makes it easier to perform controller synthesis with simplified models that reduce the nonlinearity of robot’s centroidal dynamics. In addition, the rolling joint is highly backdrivable because of its cable-driven actuation, which leads to less backlash and high torque transparency (Hwangbo et al., 2018). This also enables the robot’s knees to have a large RoM, which allows us to transport the robot more easily in comparison to other robots of similar size. It is noted that even though DRACO 3 mostly uses off-the-shelf actuators, they are all powered with Apptronik’s electronic boards via EtherCAT bus communications. The usage of multiple encoders per joint allows for more accurate control authority over the joints.

### 2.2 Hip pitch joint

The schematic diagram of the hip pitch mechanism is shown in Figure 2B. As shown in the figure, this joint is actuated by means of a rolling contact. A pair of stiff cables (cable 1 and cable 2) is routed from one end of a sheave fixed to the hip to the opposite end of a rotating sheave fixed to the motor shaft. As the shaft rotates, it wraps and unwraps the rotating sheave around the fixed sheave. In order to effectively realize the rolling contact, we remove any slack by tensioning and crimping first the non-adjustable termination (labeled (b)) and then the adjustable termination (labeled (a)). The remaining slack is then removed by unscrewing the vented screw. Since the hip motor is rigidly attached to the thigh structure, it causes the entire thigh to pivot around the hip joint, turning this into a revolute joint.

This design has taken into account several additional considerations: the cable material selection, the termination design, and the sensor placement. Typical materials for cable-driven actuators include stainless steel, Vectran, and Kevlar, among others (Hwangbo et al., 2018), where it is often desirable to have high stiffness, low creep, and low D-to-d ratio (excluding environmental resistance specifications, e.g., water resistance). Since the bending radii in our design are fairly big in comparison to the required cable diameter, the D-to-d ratio is relaxed, thus favoring stainless steel. On the other hand, terminations for stainless steel tend to be a bit more restrictive since they can occupy a fair amount of space, thus fitting them in tight spaces can become challenging. For our design, we have made custom barrel-end cables by silver soldering the cables to customized barrels and have crimped loop sleeves over vented screws on the other end, as illustrated in Figure 2B. These terminations prevent the cables from losing grip at their ends, allowing us to effectively use stainless steel cables of 1.5 mm diameter for our humanoid robot. The position of the thigh is measured with an absolute encoder located at the pivoting joint on the fixed sheave, with its corresponding magnet aligned in the thigh linkage structure.

### 2.3 Knee joint

The knee joint is designed as a rolling contact mechanism to ensure a large RoM. This not only enlarges the workspace of the robot but also increases its transportability by enabling the legs to be completely foldable. The rolling contact mechanism is realized similarly as in the hip pitch by using a tensioning cable-pair. The routing of the cables and their respective terminations are shown in Figure 2C. Similar to the hip pitch joint, in order to realize reliable rolling contacts, any slack can be removed by tensioning the adjustable terminations (labeled (c)). Unlike the hips, in this case, the pivoting point is not fixed but instead occurs at the interface of the rolling contact and is thus continuously changing. In addition, this mechanism has an adjustable tensioner (pulley tensioner as shown in Figure 2C (right)) connecting the thigh and shin links, which pushes them apart from each other, thus tensioning the belt drive to reduce backlash, and also enforces a constant distance between the two links. This mechanism fully constrains the thigh and shin links to roll without slipping (Collins, 2003) and thus forms a 1-DoF joint, as shown in Figure 2C (left). As the shin rotates, it simultaneously rotates and rolls along the end of the thigh. Since the radii of the thigh and shin rolling surfaces are identical, the absolute motion of the link w.r.t. each other is twice the rotation of the shin. For instance, *q*_knee_ = 90°, when *q*_proximal_ = 45°, due to such combined motion.

Among the different materials for cable-driven actuation mentioned in Section 2.2 , a less stiff material was chosen for the knees. Unlike the hip, the knee is kinematically constrained by more elements, thus reducing the overall tension in the cables and allowing us to use a different material instead of stainless steel. The benefit of this choice is that making the terminations for a set of cables requires no additional machinery, unlike stainless steel. For mechanical simplicity and compactness, an Estar stopper knot was used at the cable termination (Figure 2C). Although it has been reported in Hwangbo et al. (2018) that the common knot terminations in cable-driven actuation show poor performance, in our experience, the Estar stopper knot led to reliable cable terminations for the knee actuation.

**FIGURE 3:**
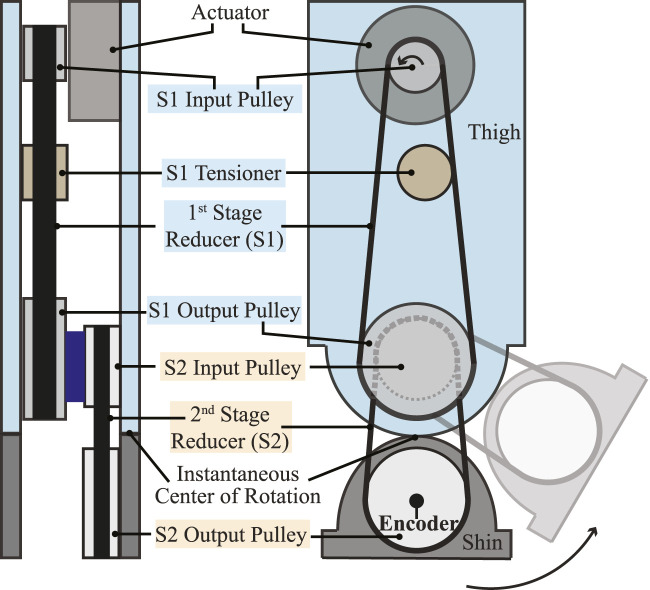
Schematic representation of the two-stage timing belt transmission. Back view (left) and sagittal view (right) of the left leg showing the transmission from the knee actuator to the knee output pulley.

Another important design consideration on the knee joint involves proximal actuation to achieve agile and dynamic motions (Sim and Ramos, 2022). The quantitative evaluation of DRACO 3’s design on proximal actuation is shown in Section 4.1.2. In DRACO 3, proximal actuation is realized by integrating a two-stage timing belt transmission, as shown in Figure 3. Mechanical power is transmitted when the motor turns the S2 output pulley via the two-stage low-loss timing belt and pulley mechanism. In addition, since the resulting transmission has lower weight and less friction than a conventional metal gearhead, it yields high torque density actuation capable of an extensive range of motion. The position of the knee is measured with an absolute encoder located at the shin linkage, as shown in Figure 3.

### 2.4 Hardware upgrade

The original humanoid robot, DRACO 3, is custom-built by Apptronik for the Human Centered Robotics Laboratory at The University of Texas at Austin. Despite being a working prototype, it has several hardware limitations: poor durability of the cable-driven mechanisms and weak actuation. These problems make it difficult for the robot to perform locomotion tasks. Therefore, we significantly upgrade the robot in all of these aspects. First, we replace the Vectran cable in hip pitch actuation with the stainless steel cable and change the knot termination to the silver-soldered custom barrel-end and the crimped loop sleeves over vented screws, as shown in Figures 2A, B. Unlike the original design, these changes substantially extend the hip joint’s durability by reducing the cable’s creeping and stretching of the terminations. Second, we upgrade the original knee motors to stronger custom-made motors, as shown in Figure 2E. Since the original motors prevent the robot from carrying its weight on the single support stance, we custom-designed the new motor with a 16:1 planetary gear ratio (which was 9:1) to meet the requirements for generating enough torque and fitting into the same tight space occupied by the original motor.

## 3 Software and control system

This section first describes the software architecture of DRACO 3 and briefly presents the state estimator used for hardware experiments. Then, a WBC formulation that accounts for the rolling contact joints is proposed, along with implementation details of the controller. In the end, a direct mapping of WBC outputs to actuator commands is described.

### 3.1 Software architecture

**FIGURE 4:**
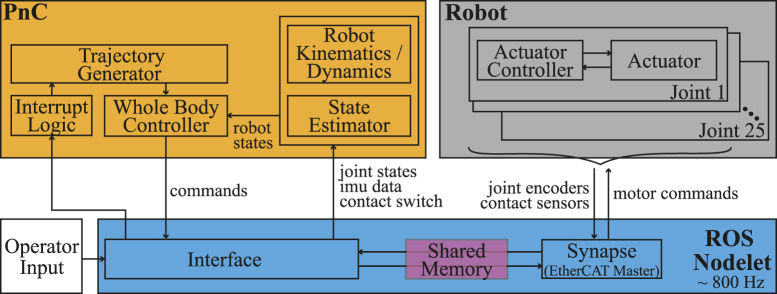
DRACO 3 software architecture. Given the user input through a ROS service call, the *Interrupt Logic* module triggers desired trajectories which are fed into WBC, leading to computing joint commands in WBC.

The control architecture of DRACO 3 consists of a decentralized controller, which runs at the actuator level, and a centralized controller, which computes the high-level commands (e.g., WBC). The interconnection between the different components of these controllers is shown in Figure 4. The DRACO 3’s control computer runs a ROS nodelet that enables communications with the robot sensors and actuators. The sensor measurements and desired control commands are then exposed using shared memory via the *Synapse* module, and we create a separate *Interface* module to package them for the high-level controller to read and write to. The high-level control actions are computed online through our previous PnC architecture (Ahn et al., 2021), which is incorporated as an external library. Given the current sensor measurements and a user command, PnC computes desired joint commands which are read by the *Interface* and sent to the *Synapse* module at the end of each synchronized control loop.

### 3.2 State estimation

Since biped or humanoid locomotion involves a sensor-based full-state feedback control and the floating base joint of the robot cannot be directly measured, it is crucial to estimate the floating base state accurately. Although there are many advanced approaches, such as using Kalman filter-based techniques (Rotella et al., 2014; Hartley et al., 2020; Camurri et al., 2020), we use a simple approach using the direct IMU and leg kinematic readings, which serves our purposes in practice. More concretely, we use IMU measurements to estimate the base joint orientation and its angular velocity and the IMU and joint encoder measurements (i.e., leg kinematics) to estimate the base linear position and velocity, respectively. The estimation of the base linear position can be estimated by keeping track of a linear offset between the contact and world frame and the forward kinematics from the base to the contact frame of a stance foot. Once the stance leg changes, the new linear offset is updated using the forward kinematics of the base to the previous and new stance positions with the old linear offset.

### 3.3 Whole-body controller

Here, we first describe our quadratic program (QP)-based WBC formulation and its constraints tailored to the rolling contact joint. We further explain how these are integrated into our QP formulation. Finally, we present the implementation details of our WBC for hardware experiments.

#### 3.3.1 QP formulation

We use the implicit hierarchical whole-body controller (IHWBC) framework from our previous work (Ahn et al., 2021), which generates smooth task and contact transitions. However, we additionally constrain our solutions to lie within the manifold of the rolling contact mechanism. The resulting optimization algorithm, formulated as QP, is written as follows:

$$\underset{\ddot{q},F_{r}}{\min}\;J\left(\ddot{q},F_{r}\right),$$ (1a)

$$s.t.\;S_{f}\left(A\ddot{q}+b+g-J_{c}^{⊤}F_{r}\right)=0,$$ (1b)

$$\;J_{\text{int}}\ddot{q}+{\dot{J}}_{\text{int}}\dot{q}=0,$$ (1c)

$$\;UF_{r}\geq 0,\;S_{r}F_{r}\leq F_{r}^{\text{max}},$$ (1d)

$$\;{\ddot{q}}_{\text{min}}\leq \ddot{q}\leq {\ddot{q}}_{\text{max}},$$ (1e)

$$\begin{array}{ll} & \;\tau_{\text{min}}\leq {\bar{S_{a}N_{\text{int}}}}^{⊤}\left(A\ddot{q}+N_{\text{int}}^{⊤}\left(b+g\right)-{\left(J_{c}N_{\text{int}}\right)}^{⊤}F_{r}\right)\leq \tau_{\text{max}}, \end{array}$$ (1f)

with

$$\begin{array}{ll} J\left(\ddot{q},F_{r}\right) & =\overset{n}{\underset{i=1}{\sum }}{\left\|J_{i}\ddot{q}+\dot{J_{i}}\dot{q}-{\ddot{x}}_{i}^{d}\right\|}_{W_{i}}^{2}+{\left\|\ddot{q}\right\|}_{W_{\ddot{q}}}^{2}\\ & \;+{\left\|F_{r}^{d}-F_{r}\right\|}_{W_{F_{r}^{d}}}^{2}+{\left\|F_{r}\right\|}_{W_{F_{r}}}^{2}, \end{array}$$

where **J**_*i*_ and ${\ddot{x}}_{i}^{d}$ are the *i*th task space Jacobian and acceleration objective, respectively, and **W**_*i*_, $W_{F_{r}^{d}}$ $W_{\ddot{q}}$ , and $W_{F_{r}}$ denote the weighting matrices for the *i*th task, reaction force, and penalties for high values of the decision variables $\ddot{q}$ . **F**_*r*_. **A**, **b**, **g**, ***τ***, **J**_*c*_, and **F**_*r*_ denote the mass/inertia matrix, Coriolis/centrifugal force, gravitational force, torque command, contact Jacobian, and reaction forces, respectively. **S**_*f*_ and **S**_*a*_ represent the floating base and actuator selection matrix, respectively. **J**_int_ and **N**_int_ are the internal constraint Jacobian and its null-space projection matrix, respectively, which will be explained in detail in the following section. **U**, **S**_*r*_, and $F_{r}^{\text{max}}$ are the contact wrench cone matrix, selection matrix used to bound the reaction force in a normal direction, and user-specified maximum force vector, respectively. The cost function penalizes errors on the task and on the reaction force trajectory tracking and includes regularization terms on the joint accelerations and reaction forces (Eq. 1a). The constraints in this QP are the floating base dynamics (Eq. 1b), rolling contact motion constraint (Eq. 1c), unilateral constraint, cone wrench constraint, maximum reaction force constraint in the normal direction (Eq. 1d), joint acceleration constraint (Eq. 1e), and torque constraint (Eq. 1f). Given the desired task specifications ${\ddot{x}}_{i}^{d}$ and desired reaction forces $F_{r}^{d}$ , the optimal joint accelerations ${\ddot{q}}^{⋆}$ and reaction forces $F_{r}^{⋆}$ are computed. The desired joint torques ***τ*** ^⋆^ are subsequently obtained using inverse dynamics, while the desired joint velocities and positions are computed by integrating ${\ddot{q}}^{⋆}$ , as described in IHMC Robotics (2018) and Jorgensen (2020). Note that in order to take into account the rolling contact joint, we have newly introduced internal constraints (Eq. 1c) and modified the actuator saturation constraints (Eq. 1f). For more details other than these changes, the reader is referred to Ahn et al. (2021).

##### 3.3.1.1 Modeling rolling contact joints

**FIGURE 5:**
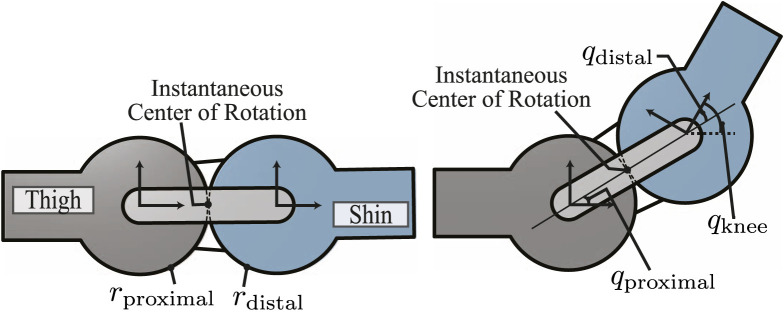
Schematics of the rolling contact joint on the knee. Zero configuration (left) and after some angular displacement (right).

The knee joint configuration is defined as the angle of the distal link (shin) with respect to the proximal link (thigh) (Collins, 2003) (Figure 5). It can be expressed as follows:

$$q_{\text{knee}}=q_{\text{proximal}}+q_{\text{distal}}=\left(1+\frac{r_{\text{distal}}}{r_{\text{proximal}}}\right)q_{\text{distal}},$$ (2)

where *q*_proximal_ and *q*_distal_ denote the proximal and distal joint positions, respectively. In particular, *q*_proximal_ = *q*_distal_ is always true in the current design. Since absolute encoders directly measuring *q*_knee_ cannot be installed due to the instantaneous center of rotation (ICR), the absolute encoder at the distal link measures *q*_distal_, which is mapped into *q*_knee_ using Eq. 2.

To integrate the kinematic model of this type of rolling contact joint into WBC, we model the 1-DoF knee joint as two revolute joints, where one is active (*q*_distal_) and the other one is passive (*q*_proximal_). This model introduces internal constraints as follows:

$$q_{\text{proximal}}=q_{\text{distal}}.$$ (3)

Based on the aforementioned relationship, we formulate the differential kinematics of the internal constraints as follows:

$$\begin{array}{cc} {\dot{x}}_{\text{int}} & =J_{\text{int}}\dot{q}=0,\\ {\ddot{x}}_{\text{int}} & =J_{\text{int}}\ddot{q}+{\dot{J}}_{\text{int}}\dot{q}=J_{\text{int}}\ddot{q}=0, \end{array}$$ (4)

where **x**_int_ ≜ **q**_distal_ − **q**_proximal_ and **J**_int_ denote the differential coordinate due to internal constraints and Jacobian for the internal constraints, respectively. In our case, the Jacobian matrix of the internal constraints is a constant matrix.

$$J_{\text{int}}=\left[\begin{array}{cccc} \underset{\text{floating base}}{\underbrace{\begin{array}{c} 0\\ 0 \end{array}}} & \underset{\text{left knee}}{\underbrace{\begin{array}{cc} \overset{\text{distal}}{\overbrace{+1}} & \overset{\text{proximal}}{\overbrace{-1}}\\ 0 & 0 \end{array}}} & \underset{\text{right knee}}{\underbrace{\begin{array}{cc} \overset{\text{distal}}{\overbrace{0}} & \overset{\text{proximal}}{\overbrace{0}}\\ +1 & -1 \end{array}}} & \underset{\text{the rest joints}}{\underbrace{\begin{array}{c} 0\\ 0 \end{array}}} \end{array}\right].$$ (5)

We utilize the aforementioned internal constraints (Eq. 4) and its Jacobian (Eq. 5) when generating a model for the robot’s constrained dynamics and formulate an optimization problem for our QP formulation.

##### 3.3.1.2 Dynamics model

Let us express the rigid-body dynamics of an *n*-DOF humanoid robot with its internal constraints as follows:

$$A\ddot{q}+b+g=S_{a}^{⊤}\tau +J_{\text{int}}^{⊤}F_{\text{int}}+J_{c}^{⊤}F_{r},$$ (6)

where $q={\left[q_{b}^{⊤},q_{a}^{⊤}\right]}^{⊤}$ , **q**_*b*_ ∈ SE(3), $q_{a}\in R^{n}$ , $\dot{q}\in R^{n+6}$ , $\ddot{q}\in R^{n+6}$ , and **F**_int_ denote the system’s configuration, unactuated position and orientation of the system’s floating base, actuated joints’ configurations, system’s generalized velocity, system’s generalized acceleration, and internal forces, respectively. Note that, in our implementation, the orientation of the floating base is parameterized through a quaternion representation, and thus the system configuration vector becomes $q={\left[p^{⊤},\varphi^{⊤},q_{a}^{⊤}\right]}^{⊤}\in R^{n+7}$ , with **p** representing the floating base position and ***φ*** representing the floating base orientation in the quaternion form. The system’s generalized velocity is represented as $\dot{q}={\left[{\dot{p}}^{⊤},\omega^{⊤},{\dot{q}}_{a}^{⊤}\right]}^{⊤}\in R^{n+6}$ , with ***ω*** representing the angular velocity. In order to optimize over $\ddot{q}$ and **F**_*r*_, we express **F**_int_ in terms of ***τ*** and **F**_*r*_ using the property ${\dot{x}}_{\text{int}}={\ddot{x}}_{\text{int}}=0$ derived in the following section as follows:

$$F_{\text{int}}=-{\bar{J}}_{\text{int}}^{⊤}\left(S_{a}^{⊤}\tau +J_{c}^{⊤}F_{r}-b-g\right)-\Lambda_{\text{int}}{\dot{J}}_{\text{int}}\dot{q},$$ (7)

where ${\bar{J}}_{\text{int}}=A^{-1}J_{\text{int}}^{⊤}\Lambda_{\text{int}}$ with $\Lambda_{\text{int}}={\left(J_{\text{int}}A^{-1}J_{\text{int}}^{⊤}\right)}^{-1}$ . Then, we substitute Eq. 7 into Eq. 6, resulting in the following constrained dynamic model:

$$A\ddot{q}+N_{\text{int}}^{⊤}\left(b+g\right)+J_{\text{int}}^{⊤}\Lambda_{\text{int}}{\dot{J}}_{\text{int}}\dot{q}={\left(S_{a}N_{\text{int}}\right)}^{⊤}\tau +{\left(J_{c}N_{\text{int}}\right)}^{⊤}F_{r},$$ (8)

where $N_{\text{int}}=I-{\bar{J}}_{\text{int}}J_{\text{int}}$ . Since the Jacobian matrix for the internal constraints is constant, the term $J_{\text{int}}^{⊤}\Lambda_{\text{int}}{\dot{J}}_{\text{int}}\dot{q}$ in Eq. 8 vanishes. In addition, it is noted that due to the structure of this Jacobian, the internal constraint force **F**_int_ does not directly affect the floating base dynamics. However, the configuration and velocity of the floating base will be indirectly driven by the actuated dynamics incorporating **F**_int_. Even though the constrained dynamics model (Eq. 8) is within the manifold of the internal constraints, Eq. 1b is not in the constrained manifold because we select only the rows of Eq. 8 corresponding to the floating base dynamics. Consequently, we explicitly include the internal constraints (Eq. 1c) in the QP formulation. Finally, the rows of Eq. 8 corresponding to the actuated joint dynamics are used to compute the torque command through inverse dynamics.

##### 3.3.1.3 Torque limits

The most intuitive way to consider the joint torque constraint is to first compute the joint torque from Eq. 6 as follows:

$$\tau =S_{a}\left(A\ddot{q}+b+g-J_{\text{int}}^{⊤}F_{\text{int}}-J_{c}^{⊤}F_{r}\right).$$

However, since our QP formulation does not optimize over the internal constraint force **F**_int_, we cannot include the aforementioned equation in our optimization problem. Therefore, we instead use the internal constraint consistent dynamics from Eq. 8 and compute the torque command as follows:

$$\tau ={\bar{S_{a}N_{\text{int}}}}^{⊤}\left(A\ddot{q}+N_{\text{int}}^{⊤}\left(b+g\right)-{\left(J_{c}N_{\text{int}}\right)}^{⊤}F_{r}\right),$$ (9)

by solving for ***τ*** in Eq. 8 with the dynamically consistent inverse of ${\left(S_{a}N_{\text{int}}\right)}^{⊤}$ , as denoted by $\bar{S_{a}N_{\text{int}}}:=A^{-1}N_{\text{int}}^{⊤}S_{a}^{⊤}{\left(S_{a}N_{\text{int}}A^{-1}N_{\text{int}}^{⊤}S_{a}^{⊤}\right)}^{†}$ and $N_{\text{int}}=N_{\text{int}}^{2}$ . {•}^†^ denotes a singular value decomposition (SVD)-based pseudo-inverse operator, in which small singular values below a user-defined threshold are set to 0. For computational efficiency, we truncate the matrices in Eq. 9 by removing the elements corresponding to the floating base. Note that Eq. 9 is only valid when $\bar{S_{a}N_{\text{int}}}S_{a}N_{\text{int}}=N_{\text{int}}$ (Lee et al., 2020), and this can be verified if we plug Eq. 9 into Eq. 8. Such property is always satisfied in our case since the internal constraint Jacobian **J**_int_ is not dependent on the configuration.

#### 3.3.2 Implementation details

Here, we discuss the practical details of our WBC implementation, including the control law and Jacobian selection for each task, as well as our choice of task weights during stance.

##### 3.3.2.1 Centroidal momentum task

Centroidal momentum control is a key task objective for humanoid locomotion. Centroidal momentum is divided into linear and angular momentum. The linear momentum task is decoupled in the horizontal and vertical components. For the horizontal components, we use the instantaneous capture point (ICP) control law (Koolen et al., 2016) as follows:

$$\begin{array}{ll} {\ddot{x}}_{{\text{CoM}}_{x,y}}^{d} & =\frac{g}{h}\left(x_{{\text{CoM}}_{x,y}}-x_{{\text{CMP}}_{x,y}}^{d},\right)\\ x_{{\text{CMP}}_{x,y}}^{d} & =x_{\text{ICP}}-\frac{g}{h}{\dot{x}}_{\text{ICP}}^{d}+k_{p}\left(x_{\text{ICP}}-x_{\text{ICP}}^{d}\right)\\ & \;+k_{i}\int \left(x_{\text{ICP}}-x_{\text{ICP}}^{d}\right)\;dt, \end{array}$$ (10)

where *g*, *h*, $x_{{\text{CMP}}_{x,y}}$ , and **x**_ICP_ denote the gravitational acceleration, the desired CoM height, the location of the centroidal moment pivot (CMP), and the ICP, respectively. The horizontal component of the CoM Jacobian is used for this task. For the vertical component, similar to Jorgensen (2020), we use the height of the base CoM link as a proxy for controlling the vertical linear momentum. In our experience, the combination of such task specifications works better than directly controlling the CoM. In addition, rather than controlling the angular momentum, we rely on torso orientation and upper body posture control to minimize the variation of angular momentum (Caron et al., 2019). In practice, we have not observed any significant performance improvement by adding the angular momentum task.

##### 3.3.2.2 Stance foot task

Kinematic contact constraints (non-slip constraints) are enforced as soft constraints in the cost function described in Eq. 1a. These constraints are given the largest weights (**W**_contact_) to reduce jerk due to the joint commands ${\ddot{q}}^{⋆}$ used for the current stance phase (Kim et al., 2020). Similar to the strategy used in Ahn et al. (2021) for the line-feet robot control, we incorporate a zero acceleration command as follows:

$${\ddot{x}}_{\text{contact}}^{d}=0,$$ (11)

with $x_{\text{contact}}^{d}=x_{\text{contact}}$ and ${\dot{x}}_{\text{contact}}^{d}={\dot{x}}_{\text{contact}}$ . In our experience, this control law significantly contributes to compliant motion especially when the swing foot touches the ground with uncertainty due to tracking errors.

In addition, for each kinematic contact constraint, we enforce a contact wrench cone constraint (Caron et al., 2015) for contact stability. To ensure smooth contact transitions, the upper bound of the normal contact force $F_{r}^{\text{max}}$ is scheduled to change depending on the contact state. Last but not the least, unlike Jorgensen (2020) and Ahn et al. (2021) that equally regularize the contact wrench **F**_*r*_ in Eq. 1a along its components with just one weighting value, we penalize the moment of ground contact forces around the local x- and *y*-axes of the contact frame more than we do for the rest of the contact wrench $W_{F_{r}}$ . This subtle change effectively regulates the center of pressure (CoP) and notably reduces the undesired motions (e.g., foot-tilting motions).

As for handling the switching of contacts, we leveraged the inequality constraint in Eq. 1d. In particular, we achieved smooth contact switching by changing the upper bound on the normal reaction forces $F_{r}^{\text{max}}$ . During the contact transition state, in the finite state machine, $F_{r}^{\text{max}}$ is selected to decrease (to zero) when the foot contacts detach from the ground and increase again when the foot makes contact.

##### 3.3.2.3 Swing foot task

Given the desired swing foot trajectories obtained using Hermite curve interpolation, we use a PD control law to compute the task space accelerations:

$$\begin{array}{ll} {\ddot{x}}_{\text{swingfoot}}^{d} & =k_{p}\left(x_{\text{swingfoot}}^{d}-x_{\text{swingfoot}}\right)\\ & \;+k_{d}\left({\dot{x}}_{\text{swingfoot}}^{d}-{\dot{x}}_{\text{swingfoot}}\right), \end{array}$$ (12)

with lower task weights **W**_swingfoot_ than the stance foot task weights **W**_contact_. However, we compute a Jacobian for the swing foot modified with zeros on the floating base joints (Jorgensen, 2020). Similar to the case of the Jacobian for the floating base used for CoM height control in Section 3.3.2.1, the resulting swing foot Jacobian effectively excludes the contributions of the floating base joints during swing motions. In particular, this modified Jacobian effectively decouples the swing foot task and the base height task, leading to the stance leg being the only contributor to changing the height of the robot. In practice, this task decomposition enables us to precisely predict which joints affect which WBC task, thus allowing for systematic gain tuning.

### 3.4 Actuation mapping

Due to the non-collocated actuation at the hip and knee joints, the torques computed by WBC presented in the previous section need to be mapped to motor commands. These desired torques are applied in a feed-forward fashion in the low-level joint controller. Here, we present the aforementioned mappings.

#### 3.4.1 Hip pitch joint

The hip pitch torque command computed by WBC is expressed with respect to the center of the fixed sheave, as shown in Figure 2B, which is where the revolute joint is defined in our model. This torque is mapped from the hip joint axis to the motor axis through an effective transmission ratio of *r*_fix_/*r*_rot_, where *r*_fix_ and *r*_rot_ are the radii of the fixed and rotating sheaves, respectively.

#### 3.4.2 Knee pitch joint

The knee torque command computed by WBC is defined in terms of the knee distal joint axis (i.e., *q*_distal_ in Figure 5) since we consider the knee proximal joint as a passive joint in our robot control model. We first map the knee distal torque (***τ***_distal_) computed by WBC to the ICR of the rolling contact. In our case, the torque applied at ICR is double the knee distal torque. This can be derived by noting that 1) the power generated from the ICR and the distal frames is the same, indicating $\tau_{\text{knee}}{\dot{q}}_{\text{knee}}=\tau_{\text{distal}}{\dot{q}}_{\text{distal}}$ , and 2) ${\dot{q}}_{\text{knee}}=2{\dot{q}}_{\text{distal}}$ in Eq. 2. Then, we use the transmission ratios to map it to the motor location. To sum up, we obtain the effective torque exerted on the ICR axis, where the output torque of the knee joint is defined as shown in Figure 3, and the effective torque is half the torque applied on the knee distal joint axis, given that *r*_proximal_ = *r*_distal_. This torque is subsequently transformed into the motor axis via the corresponding transmission ratios, in which the total speed ratio (*k*_tot_) from the actuator to the ICR of the rolling contact is calculated as (Wang et al., 2018)

$$k_{\text{tot}}=k_{1}\left(k_{2}+1\right)/2,$$ (13)

where *k*_1_ and *k*_2_ denote the speed ratio of the S1 and S2 pulley transmissions, respectively, as shown in Figure 3.

## 4 Results and discussion

In this section, we first present our evaluation of the design choices presented in Section 2.2 and Section 2.3, as well as the proximal actuation design behind DRACO 3. Then, we demonstrate the performance of the real hardware with our proposed WBC architecture. The hardware experiments were conducted after achieving such motions in simulation using PyBullet (Coumans and Bai, 2016) but are omitted here to focus on hardware details. We only include simulations of a sample scenario that showcases the extensive RoM of the robot. A video containing the hardware experiments is available at https://www.youtube.com/watch?v=9qVQzY0fic8.

### 4.1 Evaluation of the robot design

The evaluation of the design choices presented in Section 2.2 and Section 2.3 is discussed first, followed by the evaluation of the proximal actuation design. The former contains empirical results from our experiments on the robot, while the latter is based on the Centroidal Inertia Isotropy (CII) metric (Sim and Ramos, 2022). Finally, an analysis of the vertical workspace of DRACO 3 is presented to evaluate the benefit of having RCJs.

#### 4.1.1 Evaluation of design modifications

We first assess the performance of DRACO 3 when equipped with Vectran cables both on the knees and hips. This resulted in several complications, all of which were more pronounced on the hip: 1) the tensioned cable length constantly elongated due to the use of termination knots that slide over time (although this problem was mitigated with the use of Estar knots) and minor creeping of the cable, 2) mechanical failure of the cables occurred often after employing mechanical spacers to mitigate undesirable cable elongation, and 3) cable failure was exacerbated by unintentionally twisting the cable during the tensioning process, thus weakening the fibers. As a result, the Vectran cable on the hips only allowed us to use the robot for a few hours of experimentation, after which the cables showed heavy wear, especially near the cable terminations. On the other hand, our tests with the stainless steel cable on the hip (and corresponding terminations) have highly mitigated all of the aforementioned problems, which have allowed us to perform well over 200 h of experiments without having to replace them. Even though the aforementioned problems are somewhat present in the knees, they are less problematic as the tension is shared with the knee’s belt drive.

#### 4.1.2 Centroidal Inertia Isotropy analysis

We seek to quantify the mechanical improvements gained from the proximal actuation design on DRACO 3. At the same time, we illustrate how DRACO 3 compares against other adult-sized humanoid robots. As a metric to quantify the inertial contribution of limb motions, we consider the CII metric, which evaluates a system’s proximodistal mass distribution for a nominal and current joint configuration pair. In particular, for any set of test configurations, **Q**, the CII is defined as

$$\text{CII}\left(q,q_{0}\right)≔\text{det}\left(I_{G}^{-1}\left(q\right)I_{G}\left(q_{0}\right)-1_{3}\right),$$

where **q**, **q**_**0**_ ∈ **Q**, and $I_{G}\in R^{3\times 3}$ are the configuration, nominal configuration, and rotational centroidal composite rigid body inertia matrix (Orin et al., 2013), respectively.

**FIGURE 6:**
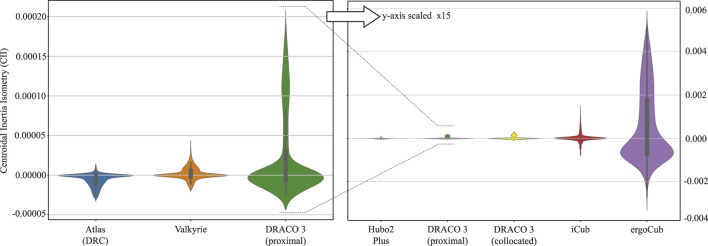
CII comparisons across various robots. Distributions of CII computed for test configurations corresponding to a single-step walking trajectory for the adult-sized humanoid robots in Kuindersma et al. (2016), Radford et al. (2015), Grey et al. (2013), Parmiggiani et al. (2011), iCub Tech IIT (2023), and DRACO 3. The left figure describes the CII comparisons with bigger and heavier robots than DRACO 3, while the right figure shows robots with a similar weight and height to DRACO 3. A large absolute value of CII means significant inertia changes compared to the nominal pose. Note that the CII was computed without considering the battery pack on each robot’s torso. For DRACO 3, the swing foot target (stepping) location varied within [0.1, 0.2] meters forward and [-0.1, 0.1] meters laterally, with a swing height of 0.05 m. A total of 30 data points are sampled along each swing trajectory. The base trajectory was sampled in the middle of the stance and swing foot.

When generating the CII values, we chose the nominal configurations such that the robots are in the upright position with their arms fully extended to the sides and their knees bent by 90°. Unlike Sim and Ramos (2022), in which the test configurations consist of motions of only the hip abduction/adduction and hip flexion/extension joints in the joint space, in order to consider motion primitives in the operational space, we computed the CII values by generating one-step motions in the Cartesian space, where the step length of each robot is normalized based on the robot’s height for a fair and practical comparison across robots. We then obtained the set of test configurations by using inverse kinematics, resulting in a set of 3,000 configurations per robot, and their associated CII values are shown in Figure 6. Note that along with DRACO 3 (proximal), we have considered as well the case of DRACO 3 (collocated), which we have created by placing the knee motor and its transmission (corresponding to 2 kg) at the knee instead of its current location. This led to three main results. First, we notice that the proximal design of DRACO 3 reduces the effect of leg inertia by 36%, as obtained by the range of CII values, which goes from 0.00033 to 0.00021. Second, among the humanoids with similar mechanical specifications (approximately 1.3–1.5 m height and 40–50 kg weight) shown on the right plot in Figure 6, DRACO 3 with proximal actuation has relatively smaller CII values than others, implying that the change in the inertia of the whole system while walking is minimal. Lastly, unlike other relatively taller and heavier humanoids such as Atlas and Valkyrie, DRACO 3 with proximal actuation has bigger CII values (on the left plot in Figure 6). This result is mostly attributed to the total leg and torso mass ratio. Although Atlas and Valkyrie have a total leg and upper body (including torso) mass ratio of approximately 40%, DRACO 3 has a ratio of 96%. In particular, even without a battery pack, the torso mass of Atlas and Valkyrie is substantially larger than that of other limbs. Therefore, to bridge the CII gap with respect to these robots, the mechatronics of DRACO 3 in the next design iteration can be altered as having a larger torso mass by adding more structural mass or relocating the motors of the other distal joints (ankle roll and pitch) near its body. Alternatively, rather than attempting to reduce the CII values by further altering its mechanical design, we allow for the gap with the other robots and consider the employment of a more descriptive model for locomotion planning and control. Specifically, since having larger CII values implies the change in the inertia of the whole system while walking is non-negligible, we can exploit the centroidal dynamics model augmented with the composite rigid body inertia proposed in Ahn et al. (2021), in which the dynamics model is used in a nonlinear trajectory optimization problem. We plan to develop an inertia-aware model predictive control (MPC) framework by adopting the dynamics model and further taking into account each limb inertia. This approach will ensure the planning result that reflects the inertia changes of the whole system, in contrast to the traditional point mass or centroidal model.

#### 4.1.3 Evaluation of RoM

**FIGURE 7:**
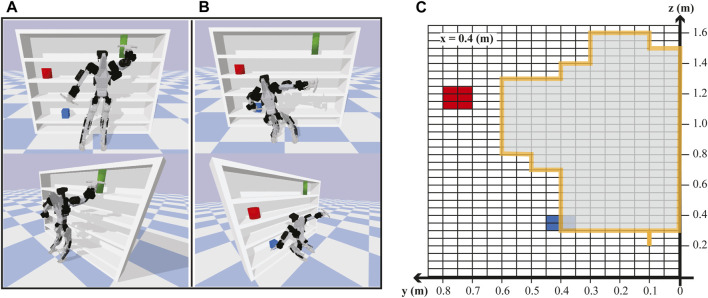
DRACO 3 hand-reaching task while balancing. Snapshots of DRACO 3 reaching the upper limit **(A)** and lower limit **(B)** of vertical postures. The reachable space (gray area) of the DRACO 3’s left hand from a nominal configuration **(C)**. Note that the torque required for each pose in the gray area is feasible given the actuator specifications.

To evaluate the extensive vertical pose workspace, which is one of the design advantages of DRACO 3, we perform a hand-reaching task while the robot balances through physics-based high-fidelity simulations in PyBullet. In particular, we study the regions where DRACO 3 with its gripper can reach objects on the bookshelf as shown in Figures 7A, B while satisfying the dynamic and kinematic constraints shown in Eq. 1. We analyze the reachable regions of the DRACO 3’s left hand shown in Figure 7C. In this study, we constrained the forward direction of the left hand to *x* = 0.4 m, discretized the space with a grid size of 0.1 m, and limited the test space for the left hand to move around the robot’s left side from its center plane. Additionally, we assumed that the position is reachable if the tracking error of each direction is within 0.03 m. From this study, the highest and the lowest height that the robot can reach with its gripper was 1.6 m and 0.2 m above the ground, respectively, with the corresponding knee angles ranging from 45° to 154°. This result demonstrates that due to the RCJs, DRACO 3 can exploit its large vertical workspace in the range of 1.4 m, which is near its height. Moreover, we analyzed the static torque required by each kinematically feasible pose in Figure 7C. For each pose, the required torque is always within the joint torque limits. From the analysis, the knee torque varied the most within different poses and reached the maximum torque of 35.33 Nm at (y, z) = (0.6, 0.8 m), which would not have been possible without the upgrade on the knee motor.

**FIGURE 8:**
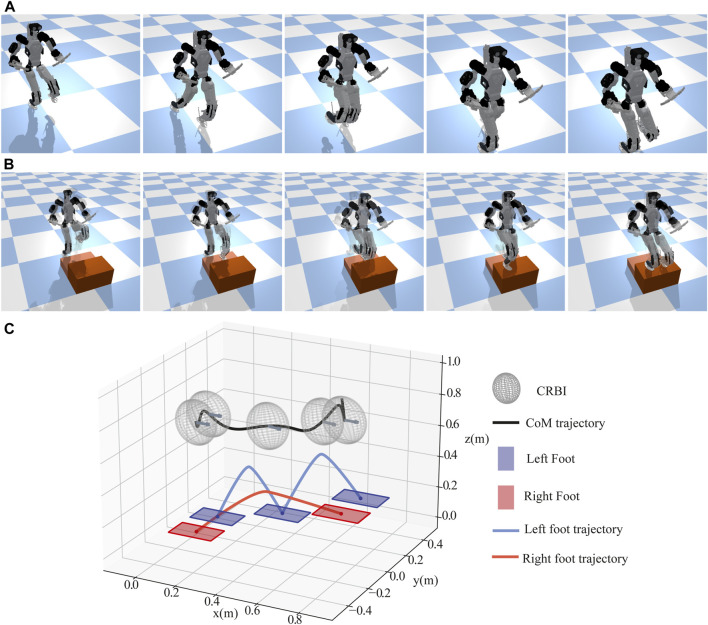
DRACO 3 locomotion tasks exploiting its RoM. Snapshots of DRACO 3 walking with big steps **(A)** and climbing stairs **(B, C)** illustrating a walking pattern for stair climbing generated from trajectory optimization.

We also study the effect of the robot’s extensive RoM on complex locomotion tasks such as walking with big steps and climbing stairs. In the walking scenario^1^ (Figure 8A), the robot can walk forward with a maximum step length of 43 cm and a swing height of 20 cm. During this motion, the robot utilizes a knee RoM ranging from 11° to 120°, which corresponds to 61% of its full RoM while stabilizing its body with the DCM planner and the proposed WBC. In the stair-climbing scenario^2^ (Figure 8B), the robot can climb up the stairs with a maximum height of 30 cm. With a walking pattern generator called TOWR+ (Ahn et al., 2021) employing the centroidal dynamics model augmented with a composite rigid body inertia (CRBI) model, we computed the trajectories of the centroidal states, feet motions, contact wrenches, and contact timing, as shown in Figure 8C. The proposed WBC then accurately tracked the trajectories while stabilizing the robot. In this locomotion task, the robot took advantage of its huge RoM by exploiting 70% of its full RoM of the knee (the left knee angle ranging from 52° to 173° and the right knee angle ranging from 29° to 156°).

#### 4.1.4 Evaluation of the rolling joint

To validate our modeling of rolling joints, we first performed a gravity compensation experiment, while the robot base was fixed, as shown in the Supplementary Video S1. In this experiment, we employed the gravity compensation control law as follows:

$$\tau ={\bar{S_{a}N_{\text{int}}}}^{⊤}N_{\text{int}}^{⊤}g,$$ (14)

which can be derived easily from Eq. 9. We verified that the rolling joint succeeded in compensating for its gravitational force and behaved as we expected. We further validated the rolling joint with an operational space control (OSC) law while commanding a sinusoidal trajectory to the left foot with an amplitude of 0.05 m and a frequency of 1 hz. The results showed that the maximum error in the tracking performance of the OSC was 0.002 m, implying that our modeling of the rolling joint in Section 3.3.1.1, the torque computation in Eq. 9, and its actuation mapping in Section 3.4.2 are accurate enough to control the joint.

**FIGURE 9:**
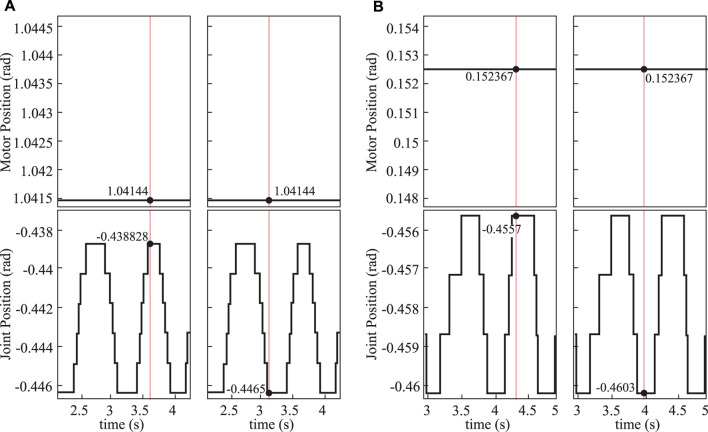
Hip pitch backlash test result. **(A)** Data for the left hip pitch motor (top) and joint (bottom) positions during the backlash test, including the maximum (left) and minimum (right) joint positions, while the motor is held in a fixed position. **(B)** Data for the right hip pitch motor (top) and joint (bottom) positions during the backlash test, including the maximum (left) and minimum (right) joint positions, while the motor is held in a fixed position. Here, the red vertical line represents the measured data for backlash calculation.

We also evaluated the backlash of the rolling joints in the hip and knee actuated by the cable-driven mechanism. In this test, we held the output (motor position) in a fixed position and captured the input (joint position) values while manually back-driving the joint. We measured the maximum and minimum joint positions, while the motor position was fixed. The difference between these maximum and minimum joint positions was considered the backlash. We obtained the backlash of the left hip pitch, right hip pitch, left knee, and right knee as 0.007677 rad, 0.004603 rad, 0.003069 rad, and 0.003069 rad, respectively, as shown in Figure 9, from which we observed the rolling joints have negligible backlash.

Although the rolling joints have the benefit of having less backlash, high backdrivability, and being distally lightweight, there are some limitations. First of all, although pretensioned to reduce elasticity, the cable employed in the transmission system is susceptible to creep no matter how stiff and strong it is. In our experience, even with the strong stainless steel and Vectran cables, we often tensioned the cable again to remove any slack due to the creep, which otherwise significantly degraded the performance of the controller. This led to frequent maintenance efforts after several experiments. Second, the cable termination is not strong enough to withstand large external impacts (i.e., when the robot steps). Oftentimes, when the robot stomps its feet during walking experiments, the termination becomes loose, leading to its reconfiguration and, eventually, replacement of the cable with a new one.

### 4.2 Hardware experiments

DRACO 3’s control PC is located off-board and runs on Ubuntu 18.04 LTS patched with the RT-Preempt kernel, which enables real-time control performance. Each actuated joint has an embedded control board for motor control and communicates with the control computer via EtherCAT. The robot is powered by an external power supply.

We performed five main hardware experiments to validate our proposed WBC implementation on DRACO 3: 1) disturbance rejection, 2) lateral swaying, 3) squatting, 4) one-leg balancing, and 5) stepping in place. All of these experiments are carried out using the same controller architecture with identical desired tasks, specifying an orientation task, feet position and orientation task, balance task through control of the ICP, upper body posture task, and regularization terms on ground reaction forces. Note that we used the implementation details described in Section 3.3.2.1, Section 3.3.2.2, and Section 3.3.2.3 throughout the experiments, and all experiments go through the states “Initialize,” “Standup,” and “Balance” before starting the actual experiments. The details of these states are described as follows:

**Initialize**: The robot is initialized in the air where the torso is supported by an overhead gantry. During this phase, all the joints are commanded to a target configuration for standing by employing a minimum jerk interpolation and the PD control law. With the help from the experimenter, the robot is placed on the ground and statically balances itself. The Initialize phase lasts for 4 s and then switches to the Standup phase.

**Standup**: The robot utilizes the ground reaction forces to lift its body to the desired base height (0.9 m). The whole-body controller performs a smooth task transition from one task set (joint posture task) to another task set (centroidal, base orientation, and left and right foot SE(3) tasks), as well as a smooth contact transition from one contact set (no contact) to another contact set (left and right foot wrench contact). Such smooth task and contact transitions are enabled by the smooth changes in the task weights and the maximum normal force of the friction cone constraints. In this phase, the desired base height trajectory is constructed using a minimum jerk interpolation, while the desired horizontal CoM is set to the middle of the left and right feet. The desired torso orientation is set to the average orientation of the two feet with quaternion SLERP. The Standup phase lasts for 1 s and then switches to the Balance phase.

**Balance**: Since WBC allows the robot to keep the CoM within its support polygon while satisfying several constraints (e.g., contact constraints and joint torque limits), the robot can balance without any support from the overhead gantry. The Balance phase lasts until receiving a user interrupt and then switches to the next phase which depends on the test scenarios.

#### 4.2.1 Disturbance rejection

**FIGURE 10:**
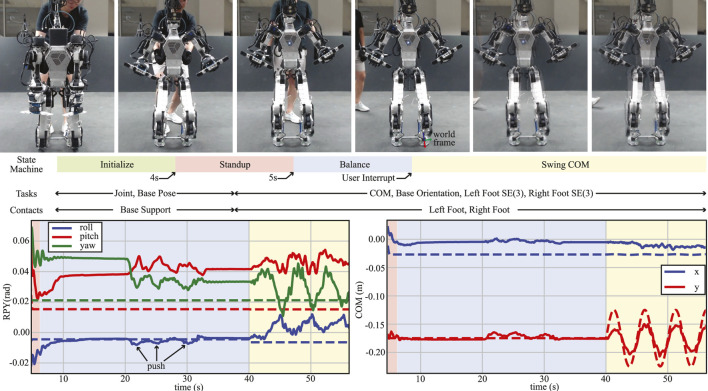
Disturbance rejection and CoM swaying test. Snapshots of the experiment as the robot progresses through the different states in the state machine (top). Desired (dashed lines) and actual (solid lines) trajectories of the roll–pitch–yaw of the torso (bottom left). Desired (dashed lines) and actual (solid lines) *x*- and *y*-positions of the CoM w.r.t. the world frame as shown in top snapshots (bottom right). In both bottom figures, the disturbances between the 20- and 30-s marks correspond to three different manual pushes performed on the robot prior to switching to the “SwingCOM” state.

The goal of this experiment is to test the balance recovery capability of our proposed WBC. In this experiment, as shown in Figure 10, we first perturbed the robot by manually performing a constant push and then an impulsive push, both in the lateral direction. The constant push moved the estimated CoM of the robot by approximately 3 cm from its desired configuration as the robot pushed against the disturbance to remain in balance. After releasing the robot from the disturbance, the robot bounced back in a direction opposite to the push and managed to stabilize again. The impulsive push was applied in a similar fashion but for a shorter time duration, to which the robot again reacted to stabilize.

**FIGURE 11:**
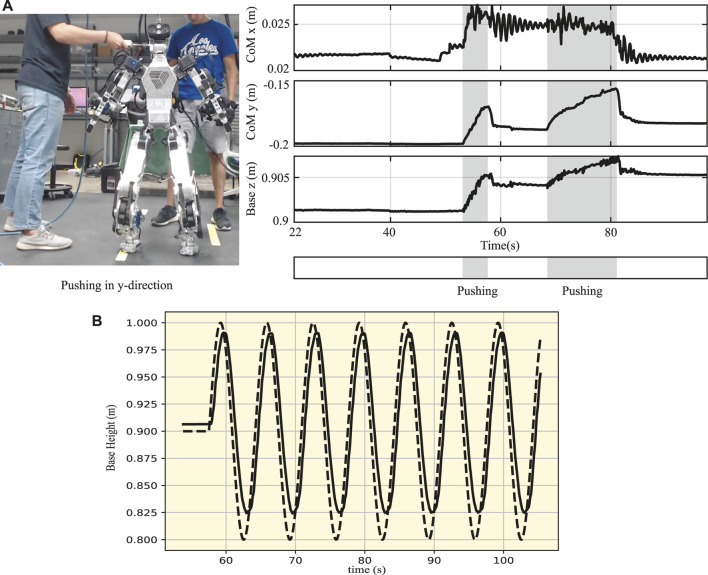
Disturbance rejection and deep squatting motion. **(A)** Constant push on the body of DRACO 3 while measuring the maximum disturbance using a digital force gauge. **(B)** Base trajectory of DRACO 3 in the vertical direction performing a 17 cm squatting motion. The reference and actual measurements are shown in dashed and solid lines, respectively.

To investigate the maximum disturbance the robot can resist, we performed two additional experiments: one involving a constant push and the other an impulsive push while measuring the external disturbance using a digital force gauge. In the first experiment, we applied a constant push at 55 s and 68 s, as shown in the marked zone (in gray) in Figure 11A. The robot was able to withstand the external disturbances and stabilize its body, during which the maximum disturbance was 34.7 N. Similarly, in the second experiment, the robot’s body was disturbed by an impulsive push (see the Supplementary Video S1), and the maximum impulsive force was 3.5 N.

#### 4.2.2 Center of mass tracking

The purpose of the following experiments is to show the ability of our WBC and overall control framework to track a non-static CoM reference while fulfilling other desired tasks.

##### 4.2.2.1 Lateral swaying

We manually triggered the robot into the “SwingCOM” state, as shown in Figure 10. In this case, the desired trajectory is a sinusoidal reference with an amplitude of 0.05 m and a frequency of 0.2 Hz, w.r.t. the world frame, which is coincident and aligned with the left foot, as shown in the fourth snapshot in Figure 10. The left graph shows the performance of the tracking of the base orientation task, indicating that the roll is tracked almost perfectly throughout this test, while the pitch and yaw remain within 1 deg of the desired pose, even during the swing motion. On the other hand, the graph on the right shows the performance of the tracking of CoM during the “Balance” and “SwingCOM” states. Although the controller is able to track the reference signal with the minimal lag, it remains 0.02 m short from tracking the 0.05 m amplitude. This is due to our current low control bandwidth in the ankle roll actuators, which we plan to improve further in future work.

##### 4.2.2.2 Squatting

A deep squatting test was carried out to evaluate the performance of the base height tracking following a sinusoidal reference trajectory, as well as to verify that DRACO 3 can perform movements requiring a large range of motion of the RCJs. Eber et al. (2021) showed that the humanoid robot RH5 was able to move its base vertically by 0.15 m in 2 s. Although shorter than RH5, we show DRACO 3 moving 0.17 m in the vertical direction by leveraging its large range of motion, as shown in Figure 11B and the Supplementary Video S1. The tracking performance demonstrates that our proposed WBC accurately modeled RCJs, thus stabilizing the robot with good accuracy. Here, we have also utilized the ICP control law as defined in Eq. 10 to control the horizontal centroidal linear momentum. It is important to note that we calculate the robot’s CoM height at every control loop, followed by the computation of the ICP and task acceleration command. This approach has proven to be effective in practice.

#### 4.2.3 One-leg balancing

**FIGURE 12:**
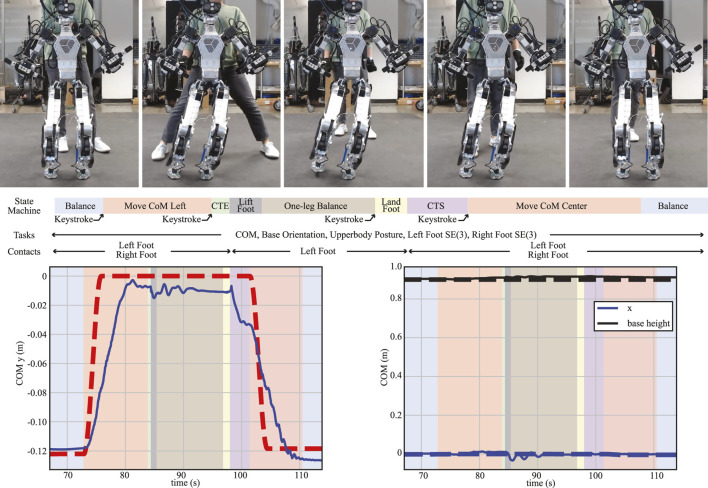
One-leg balancing. A sequence of snapshots for the one-leg balancing test is shown (top). Each corresponding state machine is described with its task and contact specifications. Desired (dashed lines) and actual (solid lines) trajectories of CoM in the lateral direction (left) and in the forward and vertical directions (right) are also plotted.

The objective of this test is to analyze the performance of the proposed WBC in a quasi-static motion, which, in this case, is comprised of a foot lift-off and a foot touchdown event. The whole sequence of trajectories generated by each state machine (Figure 12) was tracked by the proposed WBC, with each new state triggered by user interrupts (keyboard keystrokes). Each of these new states is described as follows:

##### 4.2.3.1 Move CoM Left

This state generates the new reference CoM position and velocity trajectories using the cosine interpolation method. The trajectories start from the desired values while in the “Balance” state and end at the center of the left foot. A constant desired height is commanded.

##### 4.2.3.2 ContactTransitionEnd (CTE), Lift Foot, and One-leg Balance

In this state, the contact task weights and the maximum normal contact force $F_{r}^{\text{max}}$ in Eq. 1d are linearly reduced for a smooth contact transition. Upon completion, the state machine automatically transitions to the “Lift Foot” state, which generates the swing foot trajectory using the cosine interpolation approach, given the desired height of 0.05 m. Notably, using the base and modified swing foot Jacobians presented in Section 3.3.2.1 and Section 3.3.2.3 significantly contributed to a smooth execution during this last state.

##### 4.2.3.3 Land Foot and ContactTransitionStart (CTS)

The “LandFoot” state commands the swing foot to land at the initial pose using the same interpolation method. Subsequently, contrary to the “CTE” state, the “CTS” state increases the contact task weights and the maximum normal contact force for a smooth contact transition.

##### 4.2.3.4 Move CoM Center

Similar to the “Move CoM Left” state, CoM position and velocity trajectories are generated to move the robot in 3 s from the center of the left foot position to the average position between the left and right feet.

From Figure 12, we see that despite the lag due to the relatively fast commanded motion in the “MoveCoM” states (4 m/s), the robot is able to track the CoM fairly accurately in all directions due to our WBC architecture. At the same time, it is able to stabilize when balancing on one leg during the contact changes and during the leg swing motion.

#### 4.2.4 Stepping in place

**FIGURE 13:**
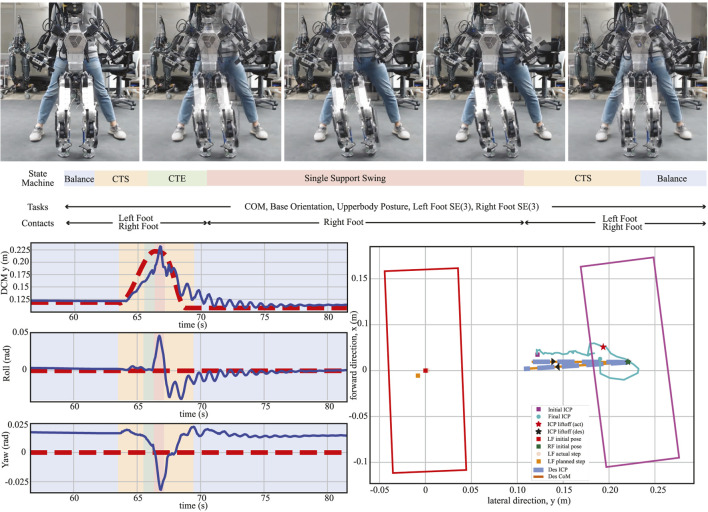
DCM-based in-place stepping. A sequence of snapshots for the DCM-based in-place stepping test is shown (top). On the left graph, the desired (dashed lines) and actual (solid lines) trajectories of CoM in the lateral direction and torso orientation in the roll and pitch directions are illustrated. Finally, the detailed information about what the DCM planner generated during this experiment is depicted on the right plot.

The tests in Section 4.2.3 focused on performing static motions. Here, we evaluated the performance of our WBC during a dynamic motion. The main difference from the one-leg balancing test is that the CoM is likely to be outside the robot’s support polygon in this test. Therefore, it is intrinsically difficult to stabilize the robot without an accurate and fast feedback controller like WBC. We used the three-dimensional DCM (Englsberger et al., 2015) to generate the trajectories for this dynamic motion. In particular, given a desired footstep sequence, the DCM planner generates desired DCM and CoM trajectories. This test consists of four different states, which are described in the following paragraphs and shown in Figure 13:

##### 4.2.4.1 ContactTransitionStart (CTS)

While similar to the “CTS” state described in Section 4.2.3, here, the CoM task trajectory is generated from the DCM planner. We commanded the robot to move its CoM toward the right (or left) foot, as shown in Figure 13, while simultaneously preparing for a smooth contact transition. We set the duration of this state to be 0.9 s. Here, the second CTS state includes additional settling time, so it was (2.2 s) longer than the first CTS.

##### 4.2.4.2 ContactTransitionEnd (CTE)

Like the “CTE” state described in Section 4.2.3, this state not only smooths the contact transition but is also used for CoM tracking, as shown Figure 13. It lasts for 0.9 s as well.

##### 4.2.4.3 SingleSupportSwing

Similar to the “Lift Foot” and “Land Foot” states described in Section 4.2.3, we set a constant desired base height and used the modified swing foot Jacobian. In addition, the Hermite curve interpolation approach was used to plan the swing foot trajectory. Here, we used a desired swing foot height and swing duration of 0.03 m and 0.8 s, respectively.

Through this DCM-based in-place stepping test, we demonstrate the effectiveness of our proposed WBC while facing several practical challenges: achieving a quasi-static motion, enabling making and breaking contacts, and swinging a distal limb with relatively high mass. In particular, as shown in Figure 13, the WBC tracked the DCM in the lateral direction with a maximum error of 0.03 m, and it tracked the torso’s roll and pitch motions with a maximum error of 0.05 rad and 0.03 rad, respectively. In our experience, the base and modified swing Jacobian contributed to reducing the error between the planned and actual footstep to within a 9 mm error.

## 5 Conclusion

This work introduced the new humanoid DRACO 3, custom-built by Apptronik, and further improved it for extended usage by the authors. One of the key components to this improvement was the material selection in cable-driven joints. We employed a combination of stainless steel and fiber cables in different parts of the robot to achieve durable actuation. We also quantified the reduced effect of leg inertia by using proximal actuation via the CII metric. Importantly, we presented a WBC that incorporates rolling contact mechanisms by considering internal constraints, provided a detailed implementation of our WBC for hardware implementation guidance, and then deployed and tested on DRACO 3 while performing disturbance rejection, lateral swaying, squatting, one-leg balancing, and in-place stepping. Our future work will include a comparison study with the conventional WBC methods (e.g., null space or HQP-based methods), as well as further experiments carrying out more complex tasks, including but not limited to walking and performing legged manipulation tasks with a more advanced state estimator (e.g., KF-based state estimator), while examining the hardware limits.

## Data Availability

The datasets presented in this study can be found in online repositories. The names of the repository/repositories and accession number(s) can be found at: https://github.com/shbang91/rpc/tree/main/test/centroidal_inertia_isometry_test, https://github.com/shbang91/PyPnC/tree/draco3_rom_test,https://github.com/shbang91/PyPnC/tree/feature/draco3-complex-motions.

## Footnotes

1 The result can be found in https://github.com/shbang91/PyPnC/tree/draco3_rom_test

2 The result can be found in https://github.com/shbang91/PyPnC/tree/feature/draco3-complex-motions
